# Make researchers revisit past publications to improve reproducibility

**DOI:** 10.12688/f1000research.12715.1

**Published:** 2017-09-21

**Authors:** Clare Fiala, Eleftherios P. Diamandis

**Affiliations:** 1 Department of Pathology and Laboratory Medicine, Mount Sinai Hospital, Toronto, ON, M5G 1X5, Canada; 2 Department of Clinical Biochemistry, University Health Network, Toronto, ON, MST 3L9, Canada; 3 Department of Laboratory Medicine and Pathobiology, University of Toronto, Toronto, ON, M5S, Canada

**Keywords:** Scientific irreproducibility, revisit past publications, reflection on past publications, improve scientific reproducibility, inflated research, bias, accountability

## Abstract

Scientific irreproducibility is a major issue that has recently increased attention from publishers, authors, funders and other players in the scientific arena.  Published literature suggests that 50-80% of all science performed is irreproducible.  While various solutions to this problem have been proposed, none of them are quick and/or cheap.  Here, we propose one way of reducing scientific irreproducibility by asking authors to revisit their previous publications and provide a commentary after five years. We believe that this measure will alert authors not to over sell their results and will help with better planning and execution of their experiments.  We invite scientific journals to adapt this proposal immediately as a prerequisite for publishing.

## Introduction

Hardly a day goes by without a screed against perverse incentives in research. It goes like this: Scientists get better rewards for announcing breakthroughs than for producing solid work. The achievements needed to win grants, jobs, and publications - combined with researchers’ (often noble) ambitions - encourage them to build castles in the air.

After that comes a plea for large-scale change. One recent proposal would require scientists to complete rigid, time-consuming confirmation studies before publishing a single paper ^1^.

We propose something that is quicker, cheaper, and simpler: Require researchers to write post-publication reflections five years after their papers appear.

In these self-reviews, researchers would assess how their claims held up. They should describe whether an invention or discovery was translated or commercialized, and how (or whether!) others could build on their work. The practice would provide a straightforward, non-stigmatized way to identify errors, misinterpretations, and other roadblocks.

For many, these-self-reviews would be a welcome opportunity for clarification, celebration, and even self-promotion. But the main advantage is that self-reviews would encourage scientists to think in advance how they might be wrong.

## Causes of irreproducibility

How might this work? Let’s consider the sources of irreproducibility. We put this down to a half-dozen causes: Often several occur together in the same paper! Fraud captures the most attention, but is rare. Self-deception, or bias, occurs aplenty. It is easier to attribute an observation to a hoped-for reason than to imagine trivial causes. Who wants to believe that a test result depends on the brand of test tube or day of the week rather than the earliest detectable sign of disease?

Then there are unrecognized technical deficiencies; researchers who know how to operate a machine, but lack enough experience to recognize artifacts and infelicities. They enter the wrong parameters or use the wrong pipette tips without realizing that they have rendered their data meaningless. Similarly, big data and data crunchers readily produce false interpretations. In 2007, one crystallographer had to retract five prominent papers after discovering a small computer glitch ^2^.

All of these problems are exacerbated by fragmented science. Projects are now executed in pieces in various laboratories and results knitted together without anyone knowing exactly what happened at each site, so no one is able to bring sufficient scrutiny to bear.

In each of these cases, the problems are clear with hindsight. If post-publication self-review was commonplace, some of these problems would become clear as experiments were being planned and conducted.

In our own lab, we have made a habit of reflecting on our papers (though not necessarily with a strict five-year timeframe). Though several papers led to work taken up by biotech companies and other scientists, others proved much less valuable than we had hoped. Bias and technical deficiencies are the most prominent reasons behind our papers that did not ‘succeed.’ That realization has made one of us a better mentor and supervisor over time. It has also led to several publications pointing out flaws in common reagents and lab practices.

Work by the psychologists Philip Tetlock and Jessica Lerner suggests that simple steps meant to hold people accountable for their judgment calls actually improves their judgment ^3^. They become more accurate in their thinking and more objective when they evaluate evidence.

Accountability in science is *ad hoc*. Researchers get credit for a publication well before enough time has passed for the scientific community to really know whether the paper has made a valuable contribution. No wonder that researchers bent on submitting a paper are obsessed with making the best possible case for its acceptance rather than illustrating its limitations. If researchers are forced to consider how well their paper will stand up five years hence, they will be more careful when doing the work and more critical in their analysis.

About ten years ago, one of us came up with the idea of a new journal, tentatively titled *Reflections in Medicin*e, in which authors of prominent papers could publish their post-publication thoughts, and contacted about 20 prospective authors, who all ignored or refused the request. We believe some did not want to revisit problematic results.

With the advent of electronic publishing, it is now possible for journals (or funders or other platforms, such as PubMed) to create a space for these five-year reflections and to connect them with the original paper.

Self-evaluation, based on strict criteria and instructions, can be revealing even if the authors try to inflate the impact of old work. For example, the boldest claims in a scientific paper should be annotated and addressed directly in authors’ reflections. Researchers could also be asked a series of straightforward yes/no questions about whether the results of a paper have changed clinical or scientific practice.

Journals, funders, or research institutions could oblige scientists to write self-reflections. Failing to do so would be a red flag. One can imagine a system in which publications in reference lists or literature databases could be annotated as lacking self-review, and so taken less seriously.

With luck, care, and enthusiasm, this simple, inexpensive step would counter perverse incentives. Instead of being stigmatized for correcting a paper, researchers would be stigmatized for failing to do so. Junior scientists would learn by example how to read papers critically and design more-rigorous experiments. The public would learn that a paper is not a definitive statement, but a single contributor to a gradually emerging picture of how nature works.

In short, self-reflections could demote scientific papers to their rightful place and turn a vicious cycle into a virtuous one.
