# Supplementary material for: Make researchers revisit past publications to improve reproducibility
Source: F1000Res. 2017 Sep 21;6:1717. [Version 1] doi: 10.12688/f1000research.12715.1 (PMC5676189; doi:10.12688/f1000research.12715.1)
Supplement: Peer review history

# Peer review: Referee response for version 1

**DOI:** 10.5256/f1000research.13773.r27534

**Published:** 2017-11-13

**Authors:** Jake Cosme

**Affiliations:** 1 Department of Laboratory Medicine and Pathobiology, University of Toronto, Toronto, ON, Canada

This review article brings forward a recommendation of mitigating the current issues of reproducibility in academic research by encouraging authors to perform post-publication self-review on their studies in 5 years. 

 The authors of the review article suggest that the implementation of self-review will allow for academic research to be undertaken with better judgment, as accountability in their work is emphasized. The value of self-review post-publication provides in principle, an excellent platform for authors to reflect on the impact and influence of their research while also providing insight on what led to possible reproducible concerns. The review article appropriately includes limitations of their proposal, such as the adoption rate by the scientific community and implementation into research programs. I believe this is an important point that should be emphasized as the value of self-review is highly dependent on the acceptance of the community. The authors insightfully recommend that self-review be included as terms for publication by "Journals, funders, and research institutions" to promote this process. 

 In summary, this is a well-written review article introducing self-reflection as a means to identify sources of irreproducibility by the authors of academic research. By encouraging self-review, peers will be able to properly place academic findings into the appropriate place in their knowledge base.

I have read this submission. I believe that I have an appropriate level of expertise to confirm that it is of an acceptable scientific standard.

# Peer review: Referee response for version 1

**DOI:** 10.5256/f1000research.13773.r26215

**Published:** 2017-11-07

**Authors:** Morley D. Hollenberg

**Affiliations:** 1 University of Calgary, Calgary, Alberta, T2N 4N1, Canada

SYNOPSIS:

 This overview deals with an enlarging key issue related to the reproducibility of published data in journals of all stripes, ranging from ‘high’ to ‘low’ impact quality.

 A ‘checkpoint’ process is proposed requiring authors to write a 5-year-post-publication ‘reflection’ on the previously published data. Failure to do so would place a ‘red flag’ beside references to that author’s work in subsequent databases and presumably also in subsequent manuscript reference sections quoting that work. The authors suggest a ‘simple’ yes/no checklist to determine whether the results of a paper have changed clinical or scientific practice.

 CRITIQUE

 This contribution is a well-written, thoughtful and concise overview of a deepening thorn in the side of published peer-reviewed data in the literature. The strength of the commentary is that the issue of long-term ‘accountability’ for those who publish the data is raised; and a ‘checkpoint’ solution is well described. The weakness in the overview is that the challenge of implementing the ‘simple questionnaire’ and the alternatives for failure of compliance are not dealt with in the text.

 For instance, one of the authors would have needed to respond to reflect on over 170 publications over the past 5 years. To determine an accurate answer about the subsequent reproducibility of the 170 findings or about the impact of the findings on clinical or basic science practice would represent a considerable challenge; and the request would be an invitation to side-step accuracy in favour of expediency to get the annoying fly off the desk. This issue is not dealt with in the text.

 Further, although author accountability is of prime importance, the suggested ‘solution’ would not surface the frustration of those e.g. other laboratories or even new trainees in the authors’ laboratories, who have difficulties repeating the published data. Those ‘negative’ findings, which are of immense importance, almost never see the light of day. The article does not deal with that key issue, which merits attention.

 SUMMARY AND RECOMMENDATION:

 This brief, well-written overview surfaces a key issue plaguing the scientific literature and offers a valuable process to enhance author accountability via a 5-year post-publication ‘reflection’ mechanism. That said, the above comments raise two issues that the authors may wish to deal with as they finalize their text. Thus, the possible outcomes of their approach (i.e. expected lack of compliance; or even worse, continued mis-representation of the data) could be dealt with and alternatives to poor outcomes for their process could be suggested. Further, a process that would enable the ‘reporting’ of lack of reproducibility from others in the field, with an opportunity for the authors to respond with ‘further reflections’ would enhance the process of accountability. As an example, this reviewer has had the opportunity to follow an enzyme isolation procedure exactly as described in a JBC manuscript; and the enzyme activity of the product was essentially as expected. That said, a biochemical sequencing of the enzyme product (NOT done in the previous JBC manuscript) showed that the enzyme was not at all the one claimed to be yielded by the published procedure. How would the ‘reflection’ process deal with that issue? One solution would be for the journal to be alerted as to the lack of reproducibility; and for the journal to insist on accountability from EACH AND EVERY ONE of those who have their names on the originally published article. The authors are encouraged to suggest an expanded ‘accountability’ process that would enhance the ‘reflection’ mechanism suggested and surface the lack of reproducibility by others of findings of published data.

I have read this submission. I believe that I have an appropriate level of expertise to confirm that it is of an acceptable scientific standard, however I have significant reservations, as outlined above.

# Peer review: Referee response for version 1

**DOI:** 10.5256/f1000research.13773.r27535

**Published:** 2017-11-06

**Authors:** Edward W. Randell

**Affiliations:** 1 Dept. of Lab. Medicine, Eastern Health Authority & Faculty of Medicine, Memorial University, St. John's, NL, Canada

The long term relevance of our published works is a point for both secret pride and disappointment for many continuing in careers with active engagement in research. This opinion article suggests that compelling researchers to reflectively evaluate their past publications and to produce a published commentary on these works (after 5 years) will improve the reliability of published work. In my opinion, this is an idea that may have reached its time and in some ways is long overdue.  While it remains to be seen if this strategy improves the quality of published work (if implemented), the user friendly and low cost proposal made by these authors at least represents a feasible approach toward improving the quality of publications by causing researchers to think twice about what they submit for publication. And this by knowing that publication involves a long term commitment and accountability to the ideas presented and to be revisited by reflective review 5 years later. Certainly, this is a well written and interesting concept that in my opinion merits consideration by journal editors and publishers, and the support of researchers committed to research excellence.

I have read this submission. I believe that I have an appropriate level of expertise to confirm that it is of an acceptable scientific standard.

# Peer review: Referee response for version 1

**DOI:** 10.5256/f1000research.13773.r26217

**Published:** 2017-09-25

**Authors:** Georgios Pampalakis

**Affiliations:** 1 Department of Pharmacy, School of Health Sciences, University of Patras, Patras, Greece

Scientific irreproducibility is indeed a serious problem nowadays. In the current opinion article, the authors state the reason that govern irreproducibility and for the first time they provide a potential method to treat such results. Importantly, their suggested method is based on a self-evaluation by the authors of a published article after a 5-year period. Indeed, this “self-review” process is simple, quick and with no additional cost. The article is very well-written for a broad audience.

I have read this submission. I believe that I have an appropriate level of expertise to confirm that it is of an acceptable scientific standard.
